# Keratinocytes Isolated From Individual Cleft Lip/Palate Patients Display Variations in Their Differentiation Potential *in vitro*

**DOI:** 10.3389/fphys.2018.01703

**Published:** 2018-11-29

**Authors:** Martin Degen, Astrid Wiederkehr, Giorgio C. La Scala, Christina Carmann, Isabelle Schnyder, Christos Katsaros

**Affiliations:** ^1^Laboratory for Oral Molecular Biology, Department of Orthodontics and Dentofacial Orthopedics, University of Bern, Bern, Switzerland; ^2^Division of Pediatric Surgery, Department of Pediatrics, Geneva University Hospitals, Geneva, Switzerland; ^3^University Clinic for Pediatric Surgery, Bern University Hospital, Bern, Switzerland

**Keywords:** cleft lip/palate, keratinocytes, differentiation, IRF6, van der Woude Syndrome

## Abstract

To gain more understanding of the complex molecular processes underlying cleft lip/palate (CLP), we established a unique human cell bank, consisting of keratinocytes and corresponding fibroblasts from individual CLP patients as a new study tool. After their careful characterization, we used such patient-derived cell cultures as well as control keratinocytes for *in vitro* differentiation and proliferation assays. Foreskin-derived control cells as a group showed significant higher induction of the late differentiation markers Loricrin and Filaggrin than the group of CLP patients-derived keratinocytes. Additionally, we detected great variations between individual CLP keratinocyte cell cultures in regard to their potential to terminally differentiate as assessed by the induction of Loricrin and Filaggrin. Primary patient cell cultures that did not properly differentiate, exhibited high proliferation rates. Moreover, we could correlate the expression levels of transcription factor *IRF6* to the ability of individual cell cultures to terminally differentiate. Using clinically relevant, patient-derived cells, our results suggest that some of the genetic predispositions causing CLP might also lead to deficiencies in keratinocyte differentiation manifested in *in vitro* assays.

## Introduction

Mature epidermis is a dynamic stratified epithelium that is constantly subject to self-renewal in a basal to superficial direction (Fuchs and Raghavan, 2002; Niemann and Watt, 2002). Mitotically active cells in the basal layer travel outward to the skin surface in a well-regulated program of terminal differentiation, which is essential for tissue homeostasis and for the acquisition of the epidermal barrier (Blanpain et al., 2007; Koster and Roop, 2007). While differentiating, keratinocytes undergo major transcriptional as well as morphological changes (Koster and Roop, 2004), which characterize the three distinct cell compartments: spinous, granular, and stratum corneum (Fuchs and Horsley, 2008). The regulation of epidermal differentiation involves the coordinated and spatiotemporal action of many different genes, repressing proliferation and triggering terminal differentiation in the suprabasal cell layers. During embryogenesis, the immature ectoderm undergoes a series of differentiation and stratification processes leading to the conversion of a single layer of undifferentiated epithelial cells to a mature epidermis (Richardson et al., 2014). The first stratification event produces a second cell layer that covers the developing epithelia with a continuous layer of flattened cells, the so-called periderm (Holbrook and Odland, 1975). The periderm acts as a barrier, prevents pathological epithelial adhesions, and persists until shortly before birth (Holbrook and Odland, 1975; Hammond et al., 2017). Any dysregulation of the balance between simple epithelial ectoderm proliferation and differentiation during development can lead to orofacial anomalies, such as cleft lip/palate (CLP) (Ingraham et al., 2006; Richardson et al., 2006; Segre, 2006).

In recent years, multiple transcription factors have been identified that control proper epidermal differentiation and barrier formation. Among them are interferon regulatory factor 6 (IRF6), grainyhead-like transcription factor 3 (GRHL3), and krüppel-like factor 4 (KLF4), which all play essential roles for the acquisition and maintenance of the periderm during embryogenesis, and that are critical for the regulation of keratinocyte proliferation/differentiation (Segre et al., 1999; Jane et al., 2005; Ting et al., 2005; Ingraham et al., 2006; Richardson et al., 2006; de la Garza et al., 2013). Distinct mutations in all three transcription factors have been described in individual CLP-affected families and patients (Kondo et al., 2002; Zucchero et al., 2004; Peyrard-Janvid et al., 2014; Leslie et al., 2016; Liu et al., 2016; Mangold et al., 2016), which underlies the importance of epidermal differentiation for proper palatogenesis (Bush and Jiang, 2012). For instance, IRF6 is found mutated in most of the van der Woude (VWS) cases, who are often characterized by lip pits and orofacial clefts (Kondo et al., 2002).

Cleft lip/palate is one of the most frequent birth defects in humans, affecting one in 500 to one in 1000 children depending on ethnic groups and geographic location (Mossey and Modell, 2012). While a lot of effort has been invested to identify genes and environmental factors responsible for the development of orofacial clefts (Meng et al., 2009), the specific genetic and/or environmental cause is only known for the minority of the cases. The complexity and frequency of this craniofacial anomaly together with the intensive and multi-disciplinary treatment approach, starting from birth until adulthood, make CLP a major psychological, social, and economic burden for CLP individuals, their parents, and society as a whole (Semb et al., 2005; Abbott et al., 2014). Hence, there is urgent need to develop novel approaches and tools to study CLP-pathogenesis and CLP-related genes in more detail on a cellular and molecular level.

We aimed to establish a human primary cell bank of keratinocytes and corresponding fibroblasts isolated from lip biopsies of CLP patients undergoing primary surgery to close the cleft lip. Since defects in the epidermal differentiation program might be the cause for a subgroup of orofacial clefts (Jiang et al., 2006), we focused our study on keratinocytes. We asked if keratinocytes isolated from individual CLP patients might display significant differences in their potential to terminally differentiate *in vitro*, when compared to each other and to control cells from healthy donors. The ideal control group for our cleft lip-derived keratinocytes would have included young children with acute lip trauma. We refrained from this option for ethical as well as practical reasons (damaged tissue, uncommon trauma in this age group). Instead, we used foreskin-derived cells for our control group as lip and foreskin cells are comparable in their tissue origin since they are both derived from a boundary between epidermis and mucosa. As this is the first report using our CLP patient-derived cells, we carefully characterized them before submitting the keratinocytes to *in vitro* differentiation assays (Hennings et al., 1980; Eckert, 1989; Bikle et al., 2012). We show that compared to control cells as a group, the induction of Loricrin and Filaggrin, markers of the outermost stratum corneum, is significantly reduced in the group of CLP patient-derived keratinocytes. Moreover, we demonstrate that individual primary patient’s cell cultures exhibit great variations in their abilities to differentiate *in vitro*. Strikingly, we learned that one of these CLP cell cultures displaying inadequate terminal differentiation, D6-Ep, originated from a clinically diagnosed VWS patient. Finally, our data suggest that the differentiation potential of CLP patient-derived keratinocytes correlates negatively with their potential to proliferate, and positively with the levels of *IRF6*, *GRHL3*, and *KLF4*.

## Materials and Methods

### Ethics Statement

This work was performed according to the Ethical Principles for Medical Research Involving Human Subjects as defined by the World Medical Association (Helsinki Declaration). Isolation of human cleft lip-, and foreskin-derived cells for this study has been approved by the Kantonale Ethikkommission of Bern, Switzerland (protocol number: 2017-01394). Written informed consent was obtained from the parents of the children.

### Cells and Cell Culture

Fresh lip tissue samples were obtained from CLP patients undergoing primary surgery at the Children’s Hospital, University of Bern to close the cleft lip at the age of 3–6 months. Clinical assessment and surgeries were performed by two highly experienced clinicians (Drs. med Schnyder and La Scala). In addition, foreskin tissue biopsies were obtained from 4 to 7 years old healthy boys (not affected by CLP) during routine circumcision at the Children’s Hospital, University of Bern.

Tissue samples were put into sterile 50 ml tubes containing approximately 20 ml of Dulbecco’s modified Eagle’s medium (DMEM; Gibco/Life Technologies; Thermo Fisher Scientific, Lucerne, Switzerland) containing 10% fetal calf serum (FCS; Seraglob, Schaffhausen, Switzerland), 1 × Pen/Strep solution (Gibco), and 1 × Amphotericin B (AmphB; Gibco). Within less than 1 h after surgery, the tissue was minced into small pieces (<1 mm^3^), placed into 6-well plates (approximately three pieces per well) in 800 μl of DMEM/10% FCS/AmphB, and placed in a humidified incubator at 37°C/5% CO_2_. Every other day, medium was carefully replenished. Explant outgrowths were observed under the microscope and cells were permitted to grow out to rather large colonies with diameters of approximately 1 cm before subculturing. In mixed cell-type outgrowths, fibroblasts were separated from keratinocytes by differential trypsinization using 0.05% trypsin-EDTA solution (Gibco) for detaching fibroblasts, followed by 0.25% trypsin-EDTA solution (Gibco) to dissociate keratinocytes. This sequential trypsinization process was attentively checked using a light microscope. From this stage, keratinocytes were cultured in keratinocyte serum-free medium (KSFM, Gibco), supplemented with 25 μg/ml bovine pituitary extract, 0.2 ng/ml epidermal growth factor, and CaCl_2_ to a final Ca^2+^ concentration of 0.4 mM, as previously described (Degen et al., 2012). To maintain healthy cells, keratinocyte cultures reaching 40% confluency were re-fed daily with 1:1 medium (1:1 vol/vol Ca^2+^-free DMEM with KSFM and supplemented as described above for KSFM alone) as described previously (Dabelsteen et al., 2009). As basal keratinocyte medium, we used KSFM supplemented as above, but without the addition of extra CaCl_2_. Hence, this medium contains only 0.1 mM CaCl_2_. The normal immortalized oral mucosal and epidermal keratinocyte cell lines, OKF6/TERT2 and N/TERT1, respectively, have been described elsewhere (Dickson et al., 2000). Fibroblasts were cultured in DMEM/10% FCS. The cells used in this study are summarized in Table 1 and Supplementary Table S1. Experiments were performed with primary cells from the second to fourth passage. All primary cells were also tested for their purities using qPCR and immunofluorescent staining. Note that each of the fibroblast and keratinocyte primary cell cultures originating from individual donors represents a mixture of fibroblasts or keratinocytes that grew out of multiple explants.

**Table 1:** Cells that have been used in this study, including the cell name, donor sex, donor age, and characteristics are indicated.

Cells	Donor sex	Donor age	Special characteristics
***ORAL KERATINOCYTES***			
Ry-Ep	M	NB	CLP
Pa-Ep	M	NB	CLP
M6-Ep	M	NB	CLP
B6-Ep	M	NB	CLP
C6-Ep	M	NB	CLP
D6-Ep	M	NB	CLP
E6-Ep	M	NB	CLP
F6-Ep	F	NB	CLP
H7-Ep	M	NB	CLP
OKF6/TERT2	M	57y	Normal; floor of the mouth; immortal
***EPIDERMAL KERATINOCYTES***
Cx-Ep	M	n.a.	Foreskin
18A-Ep	M	4y	Foreskin
18B-Ep	M	7y	Foreskin
18C-Ep	M	8y	Foreskin
18D-Ep	M	7y	Foreskin
N/TERT1	M	NB	Foreskin; immortal


*M, male; F, female; y, years; NB, newborn (between 3 and 6 months); CLP, cleft lip/palate; VWS, van der Woude Syndrome, n.a., not available.*

Prior to freezing, all primary cells were tested for mycoplasma contamination by a PCR-based mycoplasma detection assay (Praetorius, 2015) and by DAPI staining of cultures that had been grown in the absence of any antibiotics for at least 5 days.

### *In vitro* Differentiation Assays

Primary keratinocytes were thawed at passage 2 in regular KSFM growth medium. Afterwards, cultures were changed to basal KSFM medium to push them into their basal differentiation state. After 3 days in basal medium, 6 × 10^4^ keratinocytes were seeded into 35 mm tissue culture dishes for the differentiation assay in basal medium. 24 h later, CaCl_2_ was either adjusted to a final 1.8 mM (Calcium switch), supplemented with 2% FCS (FCS switch), or a combination of both to induce differentiation. At day three and five after inducing differentiation, cultures were used for further analysis.

Alternatively, for cell density-dependent differentiation, keratinocytes were grown in regular KSFM and plated into 100 mm tissue culture dishes at a cell density of 10^5^ cells. Once first colonies emerged, proteins and RNA were extracted and parallel cultures fixed for low-density (LD) analyses. Parallel cultures were re-fed every other day with KSFM, and at higher densities every day with fresh 1:1 medium. Once keratinocytes reached confluency (high-density, HD), RNA and protein were extracted and additional cultures fixed for analyses.

### Growth Assay

To assess keratinocyte growth, 2000 cells were plated in a single well of a 6-well plate (∼9 cm^2^), and counted 6–8 days later using a Neubauer Chamber. Average growth rate in terms of population doublings (PD) per day was calculated as log_2_[(number of cells obtained at subculture/number of cells plated)/number of days cultured].

### RNA Extraction, cDNA Synthesis, and Quantitative PCR (qPCR)

Total RNA was isolated from cells using the innuPREP RNA Mini kit (Analytik Jena AG, Jena, Germany) according to their standard protocol for eukaryotic cells. RNA concentration was measured and quality assessed using a Nanodrop 2000c (Thermo Fisher Scientific). RNA was stored at -80°C until use. cDNA was synthesized from 500 ng total RNA using the M-MLV Reverse Transcriptase (Promega, Dübendorf, Switzerland) and Oligo(dT)_15_ Primer (Promega). mRNA levels were quantified by qPCR using GoTaq^®^ qPCR Master Mix (Promega) on a QuantStudio 3 instrument (Applied Biosystems; Thermo Fisher Scientific). Relative RNA expression was calculated using the ΔΔ*C*_T_ method, normalizing values to GAPDH within each sample; standard error of the mean (SEM) was calculated from the results of triplicates. qPCR primers (Supplementary Table S2) were designed using the NCBI primer designing tool^1^, and tested for specificity and efficiency.

### Immunoblotting

Whole cell extracts were prepared in RIPA buffer (10 mM Tris-Cl (pH 8.0), 1 mM EDTA, 0.1% sodium deoxycholate, 0.1% SDS, 1% NP40, 140 mM NaCl) supplemented with cOmplete Mini^TM^ Protease Inhibitor cocktail and PhosSTOP EASYpack (both from Sigma-Aldrich; St. Louis, MO, United States). Protein concentrations of the lysates were measured using the BCA Protein Assay Kit (Pierce, Thermo Fisher Scientific) following their protocol. 10 μg of proteins in loading buffer (62.6 mM Tris-HCl, pH 6.8, 2% SDS, 10% glycerol, 0.01% bromophenol blue) containing 100 mM dithiothreitol (DTT) were boiled for 5 min at 95°C and separated by SDS-PAGE under reducing conditions and blotted to nitrocellulose membranes (Sigma-Aldrich). Then, membranes were stained with 0.1% amido black solution (MERCK, Schaffhausen, Switzerland) to control for equal protein loading and blotting efficiency. After blocking for 1 h at room-temperature in Tris-buffered saline (TBS, pH 7.4) containing 0.05% Tween and 5% skim milk powder (Sigma-Aldrich), membranes were incubated over-night with primary antibodies at 4°C. Membranes were washed three times in TBS-Tween and incubated for 1 h with peroxidase-conjugated anti-rabbit/mouse IgG at room-temperature. Blots were developed using SuperSignal West Dura (Thermo Fisher Scientific) and scanned by an Imager Chemi Premium Instrument (VWR, Darmstadt, Germany). Some immunoblots were analyzed densitometrically using ImageJ software version 1.51w (NIH, Bethesda, MD, United States^2^). Briefly, the intensity of each protein band was normalized to the vinculin band intensity of the same extract in the same experiment.

Primary antibodies used: Rabbit polyclonal antibodies anti-Fibronectin (Wehrle-Haller et al., 1991), anti-E-Cadherin (20874-1-AP, Proteintech, Manchester, United Kingdom), and anti-Loricrin (Thermo Fisher Scientific). Mouse monoclonal antibodies used: anti-Lamininγ2 (sc-28330, Santa Cruz, Heidelberg, Germany), anti-Involucrin (clone SY5), and anti-Vinculin (V9131, both from Sigma-Aldrich).

### Immunofluorescence

For stainings, cells were grown in 35 mm dishes containing four separate wells (Greiner Bio-One, Frickenhausen, Germany). Cultures were rinsed twice with PBS before fixation in 4% paraformaldehyde at room-temperature for 15 min. Afterwards, cells were washed three times with PBS, permeabilized in 0.1% Triton-X-100 for 5 min, blocked for 15 min in 3% BSA in TBS/0.1% Tween and incubated with primary antibody for 2 h at room-temperature. Cultures then were rinsed three times with PBS, incubated with fluorescent-labeled secondary antibodies (Molecular Probes, Thermo Fisher Scientific) or tetramethylrhodamine (TRITC)-phalloidin (Sigma-Aldrich) for 1 h light-protected, rinsed with PBS and H_2_O and coverslip-mounted with Vectashield^®^ Mounting Medium (Vector Laboratories, Burlingame, CA, United States). DAPI (Sigma-Aldrich) was added during the last washing step before mounting. Cells were examined under an Olympus BX-51 phase/fluorescence microscope (Olympus Life Science Solutions, Tokyo, Japan) equipped with a xenon lamp (X-Cite, series 120PC Q, Lumen Dynamics, Mississauga, Canada), and fluorescence filters U-MWIBA3 for AlexaFluor 488, U-MWIGA3 for Alexa Fluor 568 and TRITC, and U-MNUA2 for DAPI (Olympus Life Science Solutions). Images were captured by a ProgRes CT3 camera with ProgRes CapturePro software (Jenoptik, Jena, Germany), using either a 20×/0.5 or a 40×/0.75 NA objective. Primary antibodies are described above (see “Immunoblotting” section), except for the mouse monoclonal antibody anti-IRF6 (clone 14B2C16, BioLegend, San Diego, CA, United States).

### Tissue Sectioning and H&E Staining

Lip tissue was fixed in 4% formalin for 48 h at room-temperature. Afterwards, the fixed tissue was trimmed into appropriate size and shape and placed in embedding cassettes. The tissue was dehydrated using a series of ethanol incubations, followed by changes in xylene, before embedding tissue into paraffin blocks. 5 to 6 μm sections were cut on a Reichert-Jung microtome. Slides containing paraffin sections were deparaffinized and rehydrated through xylene, ethanol, and deionized H_2_O, stained with hematoxylin and eosin (H&E) and mounted with xylene-based mounting medium.

### Statistical Analysis

Experiments were performed in triplicates and repeated at least three times. Differences between two sets of data were statistically significant when *p* ≤ 0.05. Data are represented as means and standard deviation/standard error of the mean (SD/SEM) as stated in the figure legends. Statistical analysis using a two-tailed *t*-test was carried out at www.physics.csbsju.edu/stats/t-test.html. The Pearson correlation coefficient was calculated with the software R version 3.5.0 (The R Foundation, Vienna, Austria).

### Data Availability

The datasets generated during and/or analyzed during the current study are available from the corresponding author on reasonable request.

## Results

### Isolation of CLP-Patient Derived Cells and Their Characterization

The lack of clinically relevant human study tools to better understand the genes and their functions involved in the pathogenesis of CLP prompted us to establish a cell bank of CLP patient-derived fibroblasts and keratinocytes. During standard surgical closure of the lip, the marginal portion of the upper lip covering the cleft is in excess and needs to be removed. We collected such cleft lip tissues and initiated explant cultures to isolate primary cells.

**FIGURE 1:**
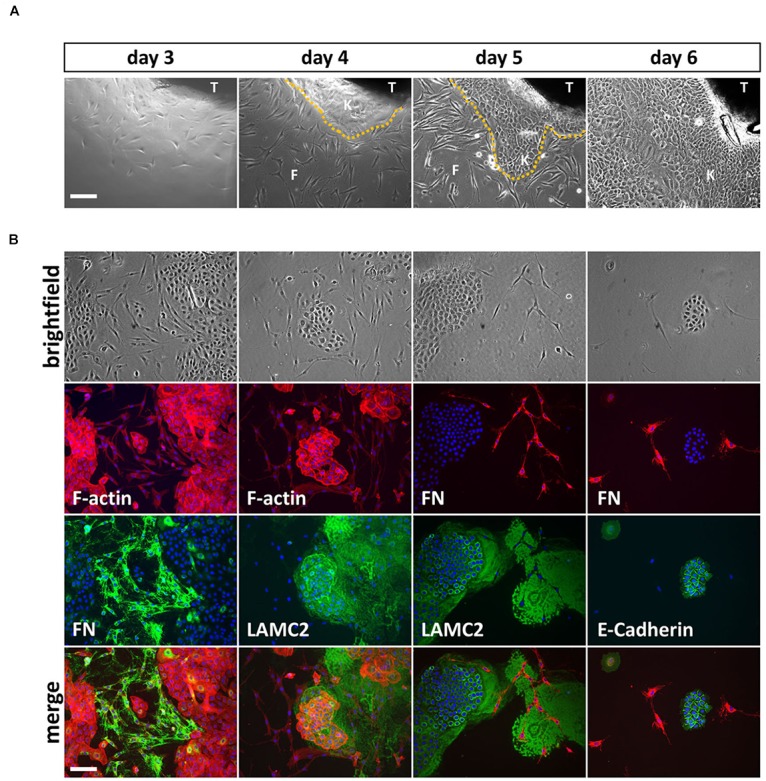
**(A)** Explant cultures and appearance of first cells growing out of the lip tissue (T) three to 6 days after initiation. Note how the cell colony is growing from day to day and the morphological differences between spindle-shaped fibroblasts (F) and the tightly packed, cobblestone-like keratinocytes (K) the dotted line represents the border between keratinocytes and fibroblasts. Scale bar: 250 μm. **(B)** Trypsinization of the cells 6 days after explant initiation often results in mixed cultures containing fibroblasts and keratinocytes. The morphological differences of the two cell types are visible in the brightfield pictures (top row) and when using phalloidin (red), which stains F-actin (second row, left two columns). Immunofluorescent stainings for a mesenchymal marker, Fibronectin (FN) and two epithelial proteins, Lamininγ2 (LAMC2) and E-Cadherin, respectively, confirms mesenchymal and epithelial origin of the cells. At the bottom, the merge of rows two and three including DAPI (blue) is shown. Note that keratinocytes secrete a large amount of LAMC2 that is deposited onto the culture dish while they move around. In contrast, FN is specifically detected where fibroblasts are located (e.g., see third column, rows two and three). Scale bar: 250 μm.

It took approximately 3 days until the appearance of the first cells growing out of the explants and within 1 week, rather big cell colonies (approximately 1 cm in diameter) surrounded the lip tissue (Figure 1A). We mostly ended up with mixed cell cultures containing different cell types (Figure 1B, top row). To determine the identity and origin of the cell types, we co-stained such mixed cultures with phalloidin (F-actin) and antibodies to either the mesenchymal-specific extracellular matrix protein Fibronectin (FN) or the epithelial basement membrane protein Lamininγ2 (LAMC2) (Figure 1B, first and second columns). The spindle-like cells stained positive for FN, indicating mesenchymal/fibroblastic identity, while the smaller and denser, cobblestone-like cells expressed LAMC2 suggesting that these cells are keratinocytes or at least of epithelial-origin. Co-stainings of cultures for FN and either LAMC2 or the epithelial adhesion marker E-Cadherin further proved exclusiveness of epithelial or mesenchymal protein expression within one cell population (Figure 1B, third and fourth columns).

**FIGURE 2:**
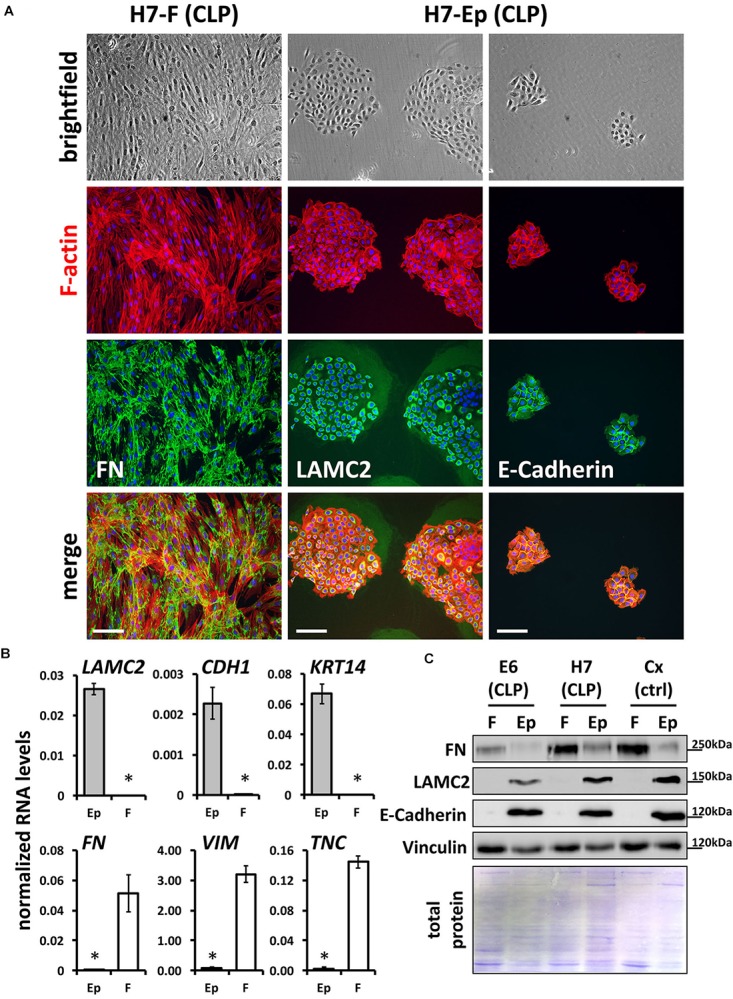
**(A)** Mixed fibroblast/keratinocyte cultures can be purified by sequential trypsinization (see section “Materials and Methods”). Note that keratinocytes and fibroblasts have completely different growth characteristics: keratinocytes grow as tightly packed colonies, whereas fibroblasts are elongated cells that grow like networks (see brightfield pictures in top row and F-actin stainings in red). In addition, immunofluorescent staining confirms cell types and their purities: fibroblast cultures are positive for their marker, FN (third and bottom rows, left column), while keratinocytes express the proteins LAMC2 and E-Cadherin (third and bottom rows, middle and right columns). Scale bar: 200 μm. **(B)** qPCR analysis for epithelial markers *Lamininγ2 (LAMC2), E-Cadherin (CDH1)*, and *Keratin 14 (KRT14)* and mesenchymal markers *Fibronectin (FN), Vimentin (VIM)*, and *Tenascin-C (TNC)*, respectively, in CLP patient-derived pure fibroblast (F) and keratinocyte (Ep) cultures. *n* = four different primary cell cultures. Data are expressed as mean ± SEM. *n* = 3. ^∗^*p* ≤ 0.05 (keratinocytes versus fibroblasts). **(C)** Immunoblot analysis of CLP patient-derived keratinocytes and fibroblasts as well as foreskin-derived control (ctrl) cells for the proteins FN, LAMC2, E-Cadherin, and Vinculin confirms identity of cells: keratinocytes (Ep) only express epithelial markers, whereas fibroblasts (F) express mesenchymal-specific proteins. Bottom panel: Amido Black staining of blotting membrane to show presence of total proteins in lysates. The blots are shown as cropped images. The full-length blots are presented in Supplementary Figure S6. kDa, kilo Dalton.

To gain pure cultures, fibroblasts and keratinocytes were differentially dissociated from culture dishes using trypsin-EDTA. All primary cells are also tested and analyzed for their purities using qPCR and immunofluorescent staining. Definitive CLP patient-derived fibroblast and keratinocyte cultures are shown in Figure 2A with the specific expression of FN by fibroblasts, and LAMC2 and E-Cadherin by keratinocytes, respectively. To confirm cell identities, we performed quantitative real-time PCR (qPCR) for three epithelial (*LAMC2*, *CDH1*, and *KRT14*) and mesenchymal (*FN, VIM*, and *TNC*) genes (Figure 2B), and carried out immunoblots for FN, LAMC2, and E-Cadherin (Figure 2C) in pure primary cultures. All these assays confirmed that we established a robust and reproducible method for the isolation of pure fibroblasts and keratinocytes from CLP patient-derived lip tissue.

### Tissue Origin of Lip-Derived Keratinocytes: Keratinized or Non-keratinized Cells?

For controls, we used foreskin biopsies, which are comparable to the lip in that both tissues represent a mucocutaneous junction area of the body. Hence, we isolated primary human keratinocytes and fibroblasts from foreskin biopsies following the described protocol (see section “Materials and Methods”).

**FIGURE 3:**
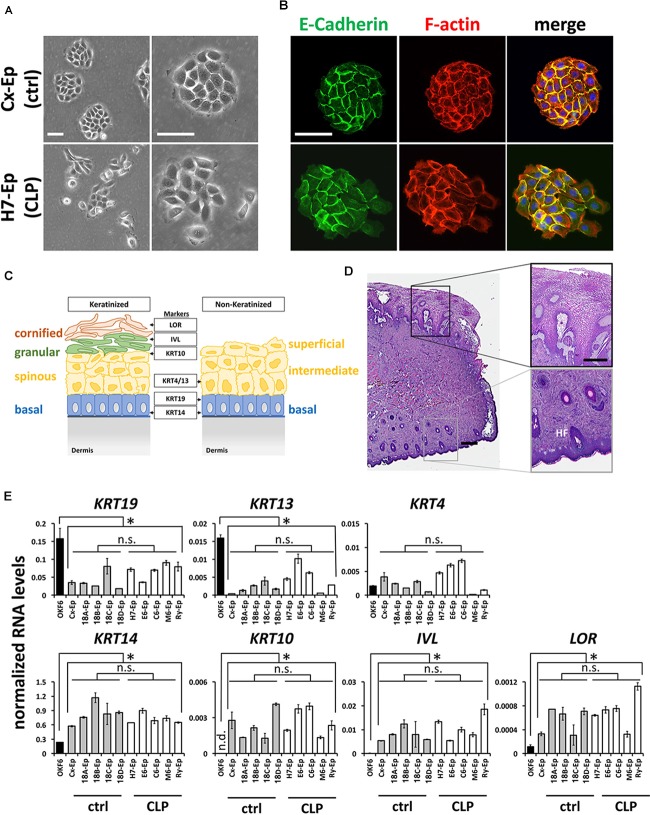
**(A)** Brightfield pictures of low density cultures of foreskin-derived control cells (Cx-Ep) and CLP patient-derived keratinocytes (H7-Ep). Scale bars: 250 μm. **(B)** Immunofluorescent staining of E-Cadherin (green) and F-actin (phalloidin, red) of low-density Cx-Ep and H7-Ep keratinocytes. Note that epidermal keratinocytes form densely packed, regular-shaped colonies, while the oral keratinocytes grow more as scattered colonies. Scale bars: 250 μm. **(C)** Schematic representation of keratinized versus non-keratinized tissue, and expression sites of markers characterizing the specific cell layers: LOR, Loricrin; IVL, Involucrin; KRT4, Keratin 4; KRT10, Keratin 10; KRT13, Keratin 13; KRT19, Keratin 19; KRT14, Keratin 14. BM, basement membrane. **(D)** H&E staining of a cleft lip biopsy (H7 Tissue) shows the region between the oral mucosa (top) and the keratinized epidermal compartment (bottom) of the infant lip. Such biopsies are used for establishing explant cultures. Scale bars: 500 μm (left); 250 μm (close-ups, right). HF, hair follicle **(E)** qPCR analysis of several differentiation markers in five foreskin-derived keratinocyte cultures (ctrl), five patient-derived keratinocyte cultures (CLP), and one oral mucosal keratinocyte cell line, OKF6/TERT2. Note that the expression of the differentiation markers in both the control as well as CLP-keratinocytes is similar to each other, but statistically different to OKF6/TERT2. Data are expressed as mean ± SEM. *n* = 3. ^∗^*p* ≤ 0.05 control- and CLP-keratinocytes versus OKF6/TERT2. n.s.: not significant.

To characterize our control group, we compared foreskin-derived to CLP patient-derived cell cultures. For example, the growth characteristics of epithelial primary cell culture H7-Ep (CLP) and Cx-Ep (control) were similar, although Cx-Ep formed more regularly shaped and cohesive colonies than H7-Ep as evidenced by light microscopy (Figure 3A) and specific stainings for F-actin and E-Cadherin (Figure 3B).

Since both the lip and foreskin are anatomical zones in which mucosa transitions to skin, we wanted to learn more about the exact tissue origin of our CLP patient-derived cells. A simplified schematic representation of keratinized and non-keratinized lip tissue is shown in Figure 3C. Histological analysis of a lip biopsy using Hematoxylin and Eosin clearly shows the mucocutaneous nature of lip tissue with co-existence of non-keratinized oral mucosa (top of the picture) as well as highly keratinized epithelium (bottom of the picture) including hair follicles (HF) and glands (Figure 3D). We concluded that our CLP-patient derived keratinocytes represent a mixture of both mucosal as well as non-mucosal cells. To confirm this notion, we tested the expression of the mucosal markers *Keratin 19* (*KRT19*) [mainly expressed in the basal layer (Lindberg and Rheinwald, 1989)] and *Keratin 4* (*KRT4*) and *Keratin 13* (*KRT13*), which are both expressed in the differentiated layers of the non-keratinized epithelium, *Keratin* *14* (*KRT14*), a marker of proliferating basal-layer cells as well as markers of keratinized, suprabasal epithelia, *Keratin 10* (*KRT10*), Involucrin (*IVL*), and Loricrin (*LOR*) (Figure 3C) by qPCR. These analyses revealed that the two primary cell groups (five CLP patient-derived cell cultures and five control cell cultures) are similar to each other, but significantly different from the oral mucosal keratinocyte cell line OKF6/TERT2 (Dickson et al., 2000), which showed highest expression of *KRT19* and *KRT13*, but lowest expression of all other markers (Figure 3E). Moreover, our final control and CLP lines did not significantly differ from each other in their proportions of mucosal and skin contribution as evidenced by the levels of *KRT4/13* (mucosa) and *KRT10* (skin) (Figure 3E).

### H7-Ep Keratinocytes Are Able to Differentiate *in vitro* Similar to Controls

**FIGURE 4:**
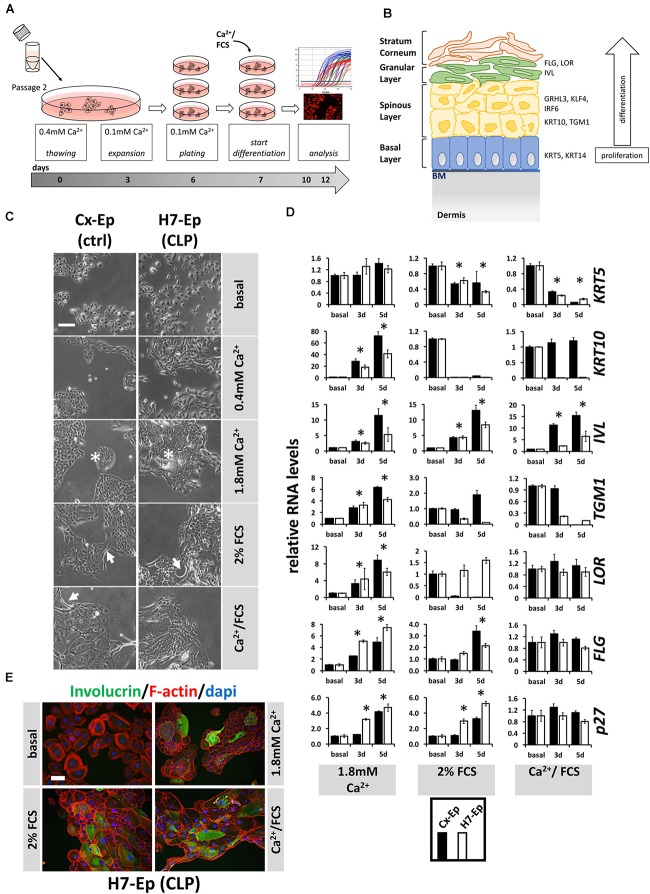
**(A)** Schematic representation of the differentiation assay used in this study. Briefly, cells at passage 2 were thawed in regular 0.4 mM Ca^2+^ KSFM. Thereafter, cells were pushed into their basal state by culturing them for 4 days in 0.1 mM Ca^2+^ KSFM before induction of differentiation for 3 or 5 days (see section “Materials and Methods”). **(B)** Schematic representation of keratinized epithelium. Shown are the four epithelial cell layers (basal, spinous, granular, stratum corneum) on the left side, and specific differentiation markers expressed in the different cell layers on the right side: markers of the basal layer: *Keratin 5* (*KRT5*), *Keratin 14* (*KRT14*); markers of the spinous layer: *Keratin 10* (*KRT10*), *Transglutaminase 1* (*TGM1*), *Interferon Regulatory Factor 6* (*IRF6*), *Grainyhead-like factor 3* (*GRHL3*), *Krüppel-like factor 4* (*KLF4*); markers of the granular layer: *Involucrin* (*IVL*), *Loricrin* (*LOR*), *Filaggrin* (*FLG*). BM, basement membrane. **(C)** Light microscopy images of control keratinocytes (Cx-Ep) and CLP keratinocytes (H7-Ep) after 5 days in differentiation media. Note that both keratinocyte cultures alter their morphology upon Ca^2+^/FCS stimuli. Asterisks: signs of stratification; Arrows: elongated cells. Scale bar: 500 μm. **(D)** qPCR analysis of various differentiation-related genes in ∼60% confluent cultures of control keratinocytes (Cx-Ep, black bars) and CLP-keratinocytes (H7-Ep, white bars) 3 and 5 days after induction of differentiation. Genes studied were: *KRT5, KRT10*, *TGM1, IVL*, *LOR*, *FLG*, and *CDKN1B* (*p27*, marker of cell cycle inhibition). Fold induction of mRNA levels is shown compared to the reference basal levels, which has been set to 1. Data are expressed as mean ± SEM. *n* = 3. Significance was reached when *p* ≤ 0.05 (^∗^) compared to basal level. **(E)** Immunofluorescent microscopy of H7-Ep CLP keratinocytes 5 days after differentiation initiation by various stimuli shows induction of the granular compartment marker Involucrin. Merged images are shown of F-actin (red), IVL (green), and DAPI (blue). Note that keratinocytes in basal medium (0.1 mM Ca^2+^) are only very loosely attached to one another. Scale bar: 250 μm.

Correct epidermal differentiation represents an important cellular mechanism for proper palatogenesis (Jiang et al., 2006). Therefore, we wondered whether all CLP patient-derived keratinocytes were able to undergo the regular differentiation program or whether the individual genotype of some primary CLP cell cultures might result in differentiation deficiencies *in vitro*. To assess this possibility, we developed our own differentiation assay (Figure 4A) and used CaCl_2_ (Ca^2+^-switch), FCS (FCS-switch), or a combination of both to induce *in vitro* differentiation (Boyce and Ham, 1983; Berghard et al., 1990; Borowiec et al., 2013). To evaluate differentiation, we analyzed a panel of markers representing the four specific epithelial cell layers during differentiation. The genes included, *Keratin 5* (*KRT5*) of the basal layer, *KRT10* and *Transglutaminase 1* (*TGM1*) of the spinous layer (early differentiation markers), *IVL*, *LOR*, and *Filaggrin* (*FLG*) of the granular layer and stratum corneum (late differentiation markers) (Figure 4B), as well as *CDKN1B* (*p27^Kip1^*), a cell cycle inhibitor. However, first, we determined the optimal Ca^2+^ concentration required to induce differentiation in our cells (Supplementary Figure S1) and decided to consistently add CaCl_2_ to a final concentration of 1.8 mM, which is in line with the literature (Borowiec et al., 2013). In an initial experiment, we selected one primary CLP patient-derived cell culture (H7-Ep) and one primary control cell culture (Cx-Ep) and subjected them to our *in vitro* differentiation assay. Five days after the addition of the distinct differentiation triggers, dramatic changes in cell morphology were evident (Figure 4C). While both keratinocyte cell cultures formed dispersed, loosely packed colonies in basal medium (0.1 mM CaCl_2_), both Ca^2+^ and/or FCS shaped tightly-packed colonies with signs of stratification in the center (asterisks), and elongated cells at the margins (arrows). To assess changes in the transcriptome in non-confluent cultures after 3 and 5 days of differentiation, we performed qPCR analyses (Figure 4E). The Ca^2+^-switch triggered a robust induction of the markers *KRT10*, *IVL*, *TGM1*, *LOR*, *FLG*, and *p27^Kip1^* in both CLP and control cells. However, presence of FCS during differentiation was required to reduce the levels of *KRT5*, which is expressed in mitotically active basal layer cells (Figure 4D). The FCS-switch elevated the levels of the late differentiation markers *IVL* and *FLG*, and of the cell cycle inhibitor *p27*, while the Ca^2+^/FCS-switch significantly raised the levels of only *IVL*. The Ca^2+^/FCS-switch also represented the harshest conditions for our primary cells, as evidenced by increasing numbers of dying cells (own observations). H7-Ep were also stained for IVL and a clear increase after 5 days of differentiation compared to low IVL levels in basal conditions could be observed confirming the qPCR results (Figure 4D). While all these data were similar in CLP and control cells in Ca^2+^-switch assays, we observed differences in the genes *KRT10*, *IVL*, *TGM1*, *LOR* between CLP and control cells under differentiation conditions containing FCS. Therefore, we used the CaCl_2_-switch assay for further studies. Importantly, the levels of the differentiation markers *KRT10* and *IVL* did not significantly increase due to higher cell density in our cultures grown in basal medium between days 3 and 5 of the assay (Supplementary Figure S2).

Additionally, we used two immortal keratinocyte lines, OKF6/TERT2 (oral mucosal) and N/TERT1 (epidermal), in our differentiation assay (Supplementary Figure S3). The outcome in these cells was very similar to that of our primary keratinocytes with some slight exceptions: in N/TERT1 and OKF6/TERT2 keratinocytes, the general morphological changes appeared to be quite subtle and proliferation arrest could not be achieved upon differentiation, and FCS was able to robustly induce late differentiation.

### Distinct Differentiation Potentials Within the Primary CLP Patient-Derived Cell Cultures *in vitro*

**FIGURE 5:**
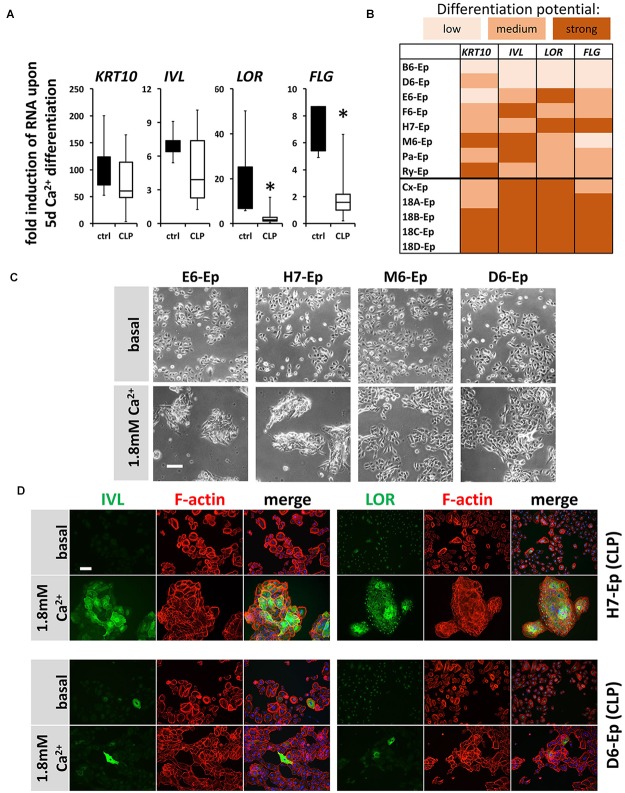
**(A)** Box plots showing the fold induction of specific genes (*KRT10*, *IVL*, *LOR*, and *FLG*) upon 5 days Ca^2+^-switch compared to their basal levels of five control keratinocyte cultures (black) and eight CLP patient-derived cell cultures (white) as assessed by qPCR analyses. Each gene in every patient line was analyzed in triplicates and the mean was used for the box plots. Note that there are statistically significant differences in the induction of the genes *LOR* and *FLG* (^∗^*p* ≤ 0.05) in control vs. CLP keratinocytes. **(B)** Heat map of Ca^2+^-induced differentiation potential of the individual keratinocyte cell cultures as assessed by qPCR analyses. Low potential: ≤50-fold induction (*KRT10*), ≤2-fold induction (*IVL*, *LOR*, *FLG*); medium potential: 50–100-fold induction (*KRT10*), 2–5-fold induction (*IVL*, *LOR*, *FLG*); strong potential: ≥100-fold induction (*KRT10*), ≥5-fold induction (*IVL*, *LOR*, *FLG*). Note that certain CLP patient-derived keratinocyte cultures, B6-Ep, D6-Ep, and M6-Ep have deficiencies in their differentiation potential *in vitro* induced by Ca^2+^-switch. **(C)** Live cell images of four CLP keratinocyte cultures grown for 5 days in 1.8 mM Ca^2+^ KSFM. Note that while the cell cultures E6-Ep and H7-Ep show clear morphological signs of differentiation, these specific morphological features are missing in the cultures D6-Ep and M6-Ep. Scale bar: 100 μm. **(D)** Immunofluorescent stainings for the proteins IVL (left panel) and LOR (right panel) in normally differentiating H7-Ep (top) and differentiation-deficient D6-Ep (bottom). While IVL and LOR are both strongly induced in H7-Ep, D6-Ep shows only minor elevation of both terminal differentiation proteins. Staining for IVL and LOR: green; F-actin: red, DAPI: blue. Scale bar: 50 μm. Note that the staining for LOR results in a nuclear background staining.

Next, we asked whether cells isolated from individual CLP patients exhibit significant variations in their CaCl_2_-induced differentiation potentials *in vitro* when compared both relative to each other and to control keratinocytes. Hence, we evaluated expression changes in the genes *KRT10*, *IVL*, *LOR*, and *FLG* 5 days after initiation of differentiation in eight CLP patient-derived and in five control cell cultures (randomly selected) by qPCR. Boxplots show that although all differentiation genes were induced in both the control and the CLP group, the levels of the late differentiation markers *LOR* and *FLG* were significantly less elevated in the CLP compared to control cells (Figure 5A). Additionally, the individual CLP patient-derived keratinocytes exhibited great variations in their ability to terminally differentiate with the primary cell cultures B6-Ep and D6-Ep (and to a lesser extent M6-Ep) having significant differentiation deficiencies as assessed by the lack of robust induction of *IVL*, *LOR*, and *FLG* (Figure 5B). These deficiencies were also visible morphologically as the cell cultures M6-Ep and D6-Ep did not show any signs of stratification as compared to the cultures E6-Ep and H7-Ep in presence of CaCl_2_ (Figure 5C). We confirmed our observations by staining for IVL and LOR in the well-differentiating H7-Ep and in the differentiation-deficient culture D6-Ep. D6-Ep clearly showed less IVL- or LOR-positive cells compared to H7-Ep upon differentiation (Figure 5D).

**FIGURE 6:**
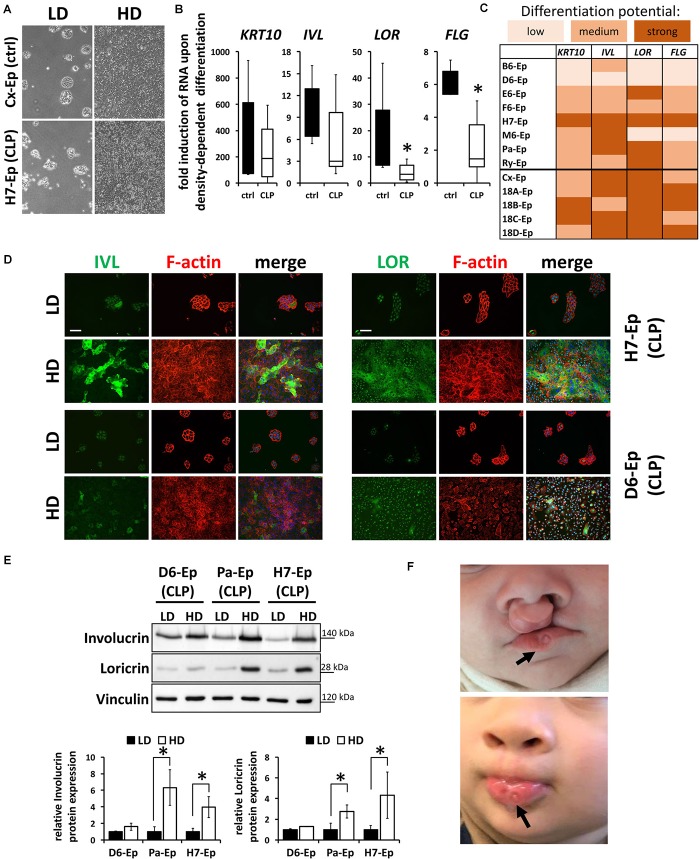
**(A)** Live cell images showing low-density (LD) and high-density (HD) cultures of control (Cx-Ep) and CLP keratinocytes (H7-Ep). Scale bar: 200 μm. **(B)** Box plots showing fold induction of the genes *KRT10*, *IVL*, *LOR*, and *FLG* upon reaching confluence (HD) compared to their levels at low-density (LD) conditions as assessed by qPCR analyses. Controls: *n* = 5 (black bars); CLP: *n* = 8 (white bars). Each gene in every patient line was analyzed in triplicates and the mean was used for the box plots. Note that there are statistically significant differences for the gene induction *LOR* and *FLG* (^∗^*p* ≤ 0.05) in control vs. CLP patient-derived keratinocytes. **(C)** Heat map of density-dependent differentiation potential of the individual keratinocyte cell cultures as assessed by qPCR analyses. Low potential: ≤100-fold induction (*KRT10*), ≤2-fold induction (*IVL*, *LOR*, *FLG*); medium potential: 100–500-fold induction (*KRT10*), 2–5-fold induction (*IVL*, *LOR*, *FLG*); strong potential: ≥500-fold induction (*KRT10*), ≥5-fold induction (*IVL*, *LOR*, *FLG*). Note that B6-Ep, D6-Ep, and M6-Ep fail to undergo terminal differentiation upon reaching confluence. **(D)** Immunofluorescent stainings for the proteins IVL (left panel) and LOR (right panel) in H7-Ep (top) and D6-Ep keratinocytes (bottom). While IVL and LOR are both strongly induced in H7-Ep, D6-Ep show defects in the induction of both terminal differentiation proteins at confluence. Staining for IVL and LOR: green; F-actin: red, DAPI: blue. Scale bar: 50 μm. Note that the staining for LOR results in a nuclear background staining. **(E)** Protein extracts of D6-Ep, Pa-Ep, and H7-Ep at low- (LD) and high-density (HD) were analyzed for Involucrin and Loricrin expression by immunoblotting (top panel). The blots are shown as cropped images. The full-length blots are presented in Supplementary Figure S6. Densitometrical quantification of the immunoblots is shown in the bottom panels. Data are expressed as mean ± SEM. *n* = 3. Note that protein levels correlate with RNA levels. **(F)** Clinical manifestation of the newborn who donated the lip tissue from which the primary cell culture D6-Ep had been derived before (top) and after surgery (bottom). Note the presence of lip pits in the lower lip (arrows), which is a diagnostic feature of van der Woude Syndrome. Written informed consent was obtained from the parents of the individual for the publication of these images.

To exclude the possibility that these data are specific for the Ca^2+^-switch, we subjected all primary cell cultures to a cell density-dependent differentiation assay (Poumay and Pittelkow, 1995). For that, we analyzed expression of *KRT10*, *IVL*, *LOR*, and *FLG* in high-density (HD) cultures and compared them to their levels in low-density (LD) cultures (Figure 6A). Confirming our Ca^2+^-switch results, *LOR* and *FLG* were significantly less elevated in the CLP group compared to the control group (Figure 6B) and the same CLP cultures, B6-Ep, D6-Ep, and M6-Ep, displayed difficulties to terminally differentiate upon density-dependent differentiation (Figure 6C). Since we did not observe any apparent differences in the proportion of oral mucosa compared to epidermal tissue between our control and CLP lines as assessed by the levels of *KRT4/13* and *KRT10* (Figure 3E), we believe that a lower induction of *LOR* and *FLG* in certain lines represents defects in their intrinsic individual differentiation potentials. These data were further supported by stainings (Figure 6D) and immunoblots for IVL and LOR (Figure 6E) in HD compared to LD cultures. These experiments emphasized the differences between the primary CLP cell cultures D6-Ep, and H7-Ep/Pa-Ep in regard to their differentiation potential *in vitro*, as assessed by the induction of IVL and LOR at HD. Prompted by these data, we carefully looked at the clinical manifestation of the newborns from whom we initially isolated the cells, to see whether *in vitro* differentiation deficiencies correlated with clinical characteristics. Strikingly, D6-Ep cells were derived from an individual who presented with a bilateral cleft and well-visible lip pits (Van Der Woude, 1954; Burdick et al., 1985), clinically diagnostic of VWS (MIM #119300) (Figure 6F). The newborns giving rise to the cells B6-Ep and M6-Ep did not present with any clinical characteristics other than CLP.

### Levels of *IRF6* Correlate Positively With the Differentiation Potential and Negatively With the Proliferation Rate of CLP Cells

**FIGURE 7:**
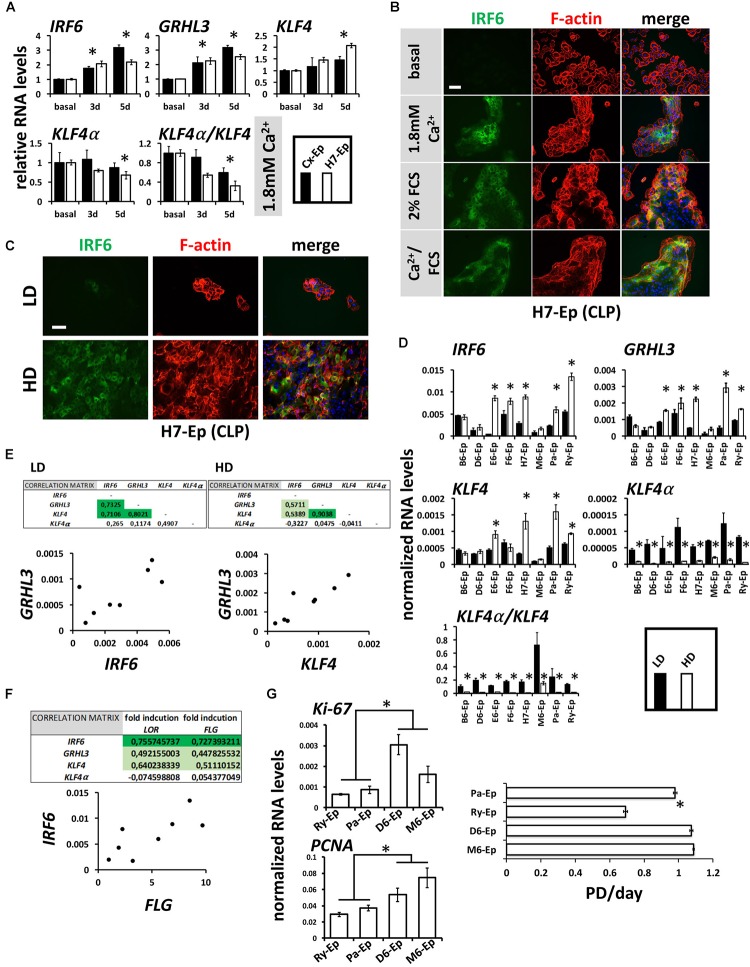
**(A)** qPCR analyses of the transcription factors *IRF6*, *GRHL3*, *KLF4* and its isoform *KLF4α* in ∼60% confluent cultures of control keratinocytes (Cx-Ep) and CLP-keratinocytes (H7-Ep) 3 and 5 days after induction of differentiation by 1.8 mM Ca^2+^. Fold induction of mRNA levels is shown compared to the reference levels (basal medium), which has been set to 1. Data are expressed as mean ± SEM. *n* = 3. Significance was reached when *p* ≤ 0.05 (^∗^) compared to basal level. While *IRF6*, *GRHL3*, and *KLF4* are induced, *KLF4α* does not drastically change upon Ca^2+^-switch. Consequently, the *KLF4α/KLF4* ratio dramatically decreases upon differentiation. **(B)** IRF6 is induced upon Ca^2+^ and FCS-induced differentiation as evidenced by immunofluorescent staining of H7-Ep. IRF6: green; F-actin: red; DAPI: blue. Scale bar: 50 μm. **(C)** Immunofluorescent analysis of H7-Ep as an example CLP cell culture that shows a prominent up-regulation of IRF6 upon reaching confluence. IRF6: green; F-actin: red; DAPI: blue. Scale bar: 50 μm. **(D)** qPCR analyses of the same three genes in CLP-keratinocytes at low-density (LD, black bars) and high-density (HD, white bars). Data are expressed as mean ± SEM. *n* = 3. Significance was reached when *p* ≤ 0.05 (^∗^). Note that certain CLP patient cultures do not induce the transcription factors upon reaching confluence. Also, *KLF4α* as well as the ratio *KLF4α/KLF4* is dramatically reduced at HD. **(E)** Correlations of the three transcription factors in LD and HD cultures within the eight CLP patient-derived keratinocyte cultures. Tables (top panels) show the Pearson’s Correlation Coefficients and an example scatter plot is shown below. **(F)** Pearson’s Correlation Coefficients (top) and scatter plot (bottom) at HD show that *IRF6* RNA levels correlate with the terminal differentiation potentials (induction of *FLG* and *LOR*) of CLP patient-derived cultures. **(G)** qPCR analysis of the proliferation markers *Ki-67* and *PCNA* in Ry-Ep, Pa-Ep, D6-Ep, and M6-Ep (left panels). The right panel shows the same four CLP keratinocytes in a growth assay. Note that higher proliferation marker expression as well highest proliferation rate was detected in those cells having problems to terminally differentiate (D6-Ep and M6-Ep). PD, Population Doublings. Data are expressed as mean ± SEM. *n* = 3. ^∗^*p* ≤ 0.05.

Mutations within the transcription factors *IRF6*, *GRHL3*, and *KLF4* are associated with clefts (Kondo et al., 2002; Zucchero et al., 2004; Peyrard-Janvid et al., 2005; Liu et al., 2016; Mangold et al., 2016). All three factors are important for keratinocyte differentiation and can be induced by CaCl_2_ in healthy human keratinocytes (Moretti et al., 2010; Hopkin et al., 2012; Sen et al., 2012). Therefore, we were interested to learn whether these transcription factors are also elevated in our CLP patient-derived keratinocyte cultures upon differentiation. CaCl_2_ was the most robust inducer of *IRF6*, *GRHL3*, and *KLF4* in H7-Ep (CLP) and Cx-Ep (control) cells (Figure 7A and Supplementary Figure S4). We also tested the levels of *KLF4α*, which is a KLF4 isoform that was recently shown to antagonize the function of KLF4 and to stimulate cancer cell proliferation (Ferralli et al., 2016). In contrast to *KLF4*, *KLF4α* was not induced under differentiating conditions, and consequently, cell differentiation resulted in a reduced *KLF4α*/*KLF4* ratio in both cell cultures (Figure 7A). Increased IRF6 levels upon *in vitro* differentiation in H7-Ep either by CaCl_2_ and/or FCS were confirmed using fluorescent microscopy (Figure 7B). IRF6 levels were also elevated upon cell density-dependent differentiation (Figure 7C). However, individual CLP cultures displayed great variation in the ability to induce levels of *IRF6*, *GRHL3*, *KLF4* at HD compared to LD (Figure 7D). Notably, the three primary CLP cell cultures B6-Ep, D6-Ep, and M6-Ep, that did not manage to undergo terminal differentiation, also showed the lowest increase of any of the three factors (Figure 7D). Within the eight individual CLP cultures, expression of *IRF6*, *GRHL3*, and *KLF4* nicely correlated with each other at LD and HD (Figure 7E and Supplementary Figure S5), and *IRF6* levels correlated with the *in vitro* differentiation potential of the same cell cultures as assessed by the fold induction of *LOR* and *FLG* upon reaching confluence (Figure 7F). Strikingly, all primary CLP cell cultures exhibited a prominent reduction of *KLF4α* and *KLF4α/KLF4* at HD compared to LD (Figure 7D).

Lastly, we compared the proliferative capabilities of Ry-Ep and Pa-Ep as two CLP cell cultures with normal differentiation potential and reasonable levels of *IRF6* to D6-Ep and M6-Ep cultures, both exhibiting problems to terminally differentiate and low *IRF6* levels. Two well-established proliferation markers (*Ki-67, PCNA*) showed reduced expression in the primary cell cultures Ry-Ep and Pa-Ep compared to D6-Ep and M6-Ep (Figure 7G, left) and accordingly, cultures D6-Ep and M6-Ep displayed higher growth rates compared to Ry-Ep and Pa-Ep (Figure 7G, right).

## Discussion

In our study, we aimed to build a human CLP cell bank allowing us to study CLP *in vitro*. So far we collected 23 lip biopsies, from which keratinocytes and fibroblasts had been isolated (Supplementary Table S1). We developed a very robust and reproducible explant culture assay for the isolation of lip tissue-derived cells. We undertook every effort possible to reduce any inter-experimental variations during primary cell isolation: tissues were processed within less than 1 h after biopsy, explant cultures were performed by the same person (MD), and the protocol (see section “Materials and Methods”) was strictly followed. In addition, all tissue donors were newborns, 3–6 months of age, when undergoing the first surgery to correct the cleft lip. Hence, we are convinced that the differences in gene expression or phenotypes within the individual patient cell cultures reflect each individual’s specific genetic predisposition rather than a culture artifact or differences in age. We used foreskin-derived cells as our control group, although we are well aware of the fact that such cells are not the best controls for our study. Analyzed parameters might be affected by differences in age and sex of the tissue donors, as well as by the different origin of tissues. We considered to use biopsies from children with acute lip trauma as control, but refrained from this possibility for ethical as well as practical reasons (damaged tissue, uncommon trauma in this age group). Our control cells may not be the ideal control, but we consider them the best compromise in a research project using human samples. Both lip and foreskin cells are comparable in their tissue origin since they are both derived from a mucocutaneous junction area of the body. Indeed, our control keratinocytes isolated from the foreskin of healthy boys displayed similar gene profiles as the CLP cultures (Figure 3), independent of the fact that they were derived from older tissue donors. Foreskin-derived cells therefore appeared to be an acceptable control group for our study. One option to further validate our control group would be to retrieve foreskin samples from the CLP group. Having such cells would allow us to demonstrate that the differences described here between CLP lip cells and healthy foreskin cells are also detectable when comparing healthy and CLP patient-derived foreskin cells. However, this was not possible since none of our male patients from the CLP group had a need for circumcision.

*In vitro* keratinocyte differentiation has been investigated for a long time. However, there is no consensus for studying this process, which makes comparisons between different analyses very difficult. Experimental parameters that add to the complexity include the use of diverse keratinocyte cells (HaCaT, mouse/human keratinocytes), the advances of the culture methods from the application of a fibroblast-feeder layer (Rheinwald and Green, 1975) to monolayered cultures, a developing variety in culture media (KSFM, EpiLife, or FCS-containing medium), different timing of differentiation, or even inconsistent culture temperatures (Borowiec et al., 2013). Although Ca^2+^ has been established as one of the main factors regulating keratinocyte differentiation (Hennings et al., 1980), a recent study indicates that presence of FCS might be beneficial for optimal keratinocyte differentiation (Borowiec et al., 2013). In our hands, the addition of FCS did not induce the expression of late differentiation markers (*FLG*, *LOR*) more efficiently than a simple single Ca^2+^-switch (Figure 4). However, presence of FCS was required for the downregulation of *KRT5*, which is in agreement with the mentioned study (Borowiec et al., 2013). Generally, FCS in the culture medium was not very well tolerated by our primary cells. For this reason and the fact that the Ca^2+^-switch was enough to induce late differentiation genes as well as an anti-proliferative gene (*p27^Kip1^*), we decided to perform all our comparative differentiation studies using CaCl_2_. Anyhow, we consider the quality of the primary cells, the time they had been cultured in basal medium and were allowed to differentiate as important as the proper differentiation trigger for robust induction of terminal differentiation. We believe that comparative studies in regard to differentiation require a strictly followed protocol including the identical passage numbers of the keratinocytes. Also, we complemented our Ca^2+^-switch data with a second CaCl_2_-independent *in vitro* differentiation assay: cell density-dependent differentiation (Figure 6). Strikingly and reassuringly, both assays resulted in the same outcomes: CLP cell cultures B6-Ep, D6-Ep, and M6-Ep exhibited deficiencies to terminally differentiate as judged by the lack of significant induction of *LOR* and *FLG* (Figures 5, 6).

While it has been known that KLF4 is up-regulated under differentiating conditions (Cordani et al., 2011; Sen et al., 2012), nothing is reported yet on the regulation of KLF4α during keratinocyte differentiation. KLF4α is one of the main isoforms of KLF4 and was found to be over-expressed in pancreatic cancer and to correlate with poor patient prognosis (Wei et al., 2010). More recently, it was shown that KLF4α is able to antagonize the function of KLF4 in breast cancer and that an increased ratio of KLF4α/KLF4 induced cancer cell proliferation (Ferralli et al., 2016). Here, we show for the first time that *KLF4α* as well as the ratio *KLF4α/KLF4* drastically decrease under differentiating conditions in primary normal and CLP keratinocytes (Figure 7). Hence, the function of the pro-differentiation factor KLF4 cannot be antagonized by low levels of the pro-proliferative KLF4α during keratinocyte differentiation.

Using a randomly selected set of eight CLP patient-derived keratinocyte cultures, we tested the hypothesis that the cause for CLP in some patients might result from gene mutations affecting keratinocyte differentiation during development, which is reflected by differentiation deficiencies *in vitro* later on using their cells. We used eight CLP patient-derived cultures since we believed that this number represented a reasonably sized set of samples that could be analyzed within a relatively short time frame to avoid experimental variations (e.g., different batch of media). Indeed, we identified B6-Ep, D6-Ep, and M6-Ep as CLP patient-derived cultures that showed defective potential to terminally differentiate (Figures 5, 6). Although we do not have any information about the CLP-associated genes in our tissue donors, we speculate that a genetic predisposition might be responsible for the development of CLP as well as for the differentiation deficiencies *in vitro* in the corresponding cells. Non-syndromic CLP remains a complex craniofacial anomaly with an unclear genetic etiology (Mehrotra, 2015). Numerous CLP candidate genes have been identified, which can be categorized into transcription factors, growth factors, extracellular matrix proteins, genes involved in metabolism, immune response, and detoxification processes (Stuppia et al., 2011). Recently, non-syndromic CLP-causing variants have been identified in genes encoding proteins responsible for the assembly of the epithelial cadherin-catenin complex. Among them are the genes *CDH1* and *CTNND1* (Brito et al., 2015; Cox et al., 2018). While pathological mutations within *CTNND1*, which encodes for the critical E-Cadherin binding partner p120^Ctn^, disrupt the E-Cadherin-p120^Ctn^ interaction, *CDH1* variants affect the extracellular calcium chelating hinge domains of E-Cadherin (Cox et al., 2018). Hence, mutations within both of these genes result in weaker or dysfunctional epithelial adhesion complexes (Cox et al., 2018). It is well established that differentiating epidermal cells require intact cell-cell junctions (Fuchs, 1990) and that interfering with the function of E-Cadherin in keratinocytes results in differentiation deficiencies (Wheelock and Jensen, 1992; Young et al., 2003; Charest et al., 2009). Hence, presence of non-syndromic CLP-causing mutations within genes of the epithelial adhesion complex (e.g., *CTNND1*, *CDH1*) might result in keratinocyte differentiation defects as we have described in our study. Recently, *CTNND1* and *CDH1* variants have also been identified as the cause of the rare Blepharocheilodontic (BCD) syndrome (OMIM 119580), which is characterized by CLP (Ghoumid et al., 2017). In this regard, it is noteworthy that after initially assuming that the tissue donors were all non-syndromic CLP patients and identifying D6-Ep as a patient cell culture having problems to terminally differentiate, we were subsequently informed that the donor of these cells presents lip pits associated with a bilateral CLP, which is a clinical diagnostic criterion for VWS.

VWS is the most common syndromic form of CLP and most of the causal VWS mutations occur within the transcription factor IRF6. IRF6 regulates the balance between keratinocyte proliferation/differentiation and *Irf6-*deficient mice display a hyperproliferative epidermis (Kondo et al., 2002). Although Irf6 is not necessary for early differentiation, Irf6 knockout mice fail to undergo terminal differentiation (no expression of Flg and Lor) leading to aberrant craniofacial morphogenesis, such as oral adhesions and clefts (Ingraham et al., 2006; Richardson et al., 2006). Using skin from the hip region of CLP children, Hixon et al., showed that the proliferation rate of VWS keratinocytes was increased when compared to non-syndromic CLP keratinocytes, both *in vivo* and *in vitro* (Hixon et al., 2016). These observations are in agreement with our results using D6-Ep keratinocytes: they failed to terminally differentiate (Figures 5, 6), but showed higher rates of proliferation than most other non-syndromic CLP cell cultures (Figure 7). Hence, the fact that the donor of the D6-Ep culture is a VWS patient supports all our results using D6-Ep keratinocytes.

Independent of VWS, we present evidence that *IRF6* levels *per se* correlate with *GRHL3* and *KLF4*, and with the differentiation potential of the individual CLP cultures (Figure 7). Since both *GRHL3* and *KLF4* have been shown to be directly regulated by IRF6 (de la Garza et al., 2013; Liu et al., 2016), a positive correlation of these three transcription factors in CLP patient-derived cells makes sense. However, we are careful with the interpretation of our results, since our correlation analysis only contained eight individual CLP cell cultures. Clearly, many more individual CLP cultures need to be analyzed in order to get a better understanding of potential correlations between *IRF6*, *GRHL3*, and *KLF4*. Nevertheless, our analysis so far clearly argues in favor of *IRF6* correlating with *GRHL3*, and *KLF4* in CLP patient-derived keratinocyte cultures *in vitro*. Moreover, qPCR analyses of the CLP patient-derived keratinocytes revealed that IRF6 levels in the VWS culture D6-Ep, as well as in the other two differentiation-deficient cultures, B6-Ep and M6-Ep, were decreased compared to all other cell cultures at HD (Figure 7D). If all three patients had mutations within *IRF6*, these results would be in agreement with the fact that haploinsufficiency is observed in CLP cases caused by IRF6 mutations. Reduced *IRF6* mRNA levels could result from gene mutations affecting *IRF6* RNA stability or by the fact that *IRF6* is a direct IRF6 target gene itself (Botti et al., 2011). Accordingly, the three primary cell cultures B6-Ep, D6-Ep, and M6-Ep also displayed the lowest RNA levels of *GRHL3* and *KLF4* among all CLP-derived cultures (Figure 7D). However, to draw any definitive conclusions, future studies should include Next-Generation-Sequencing analyses of these CLP patient-derived cell cultures.

In summary, we have established a unique and novel human CLP patient-derived cell bank of keratinocytes and corresponding fibroblasts that were isolated from discarded lip tissue obtained during the first corrective surgery of the cleft lip. We extensively characterized both the primary patient-derived fibroblasts and keratinocytes. While this study focused on keratinocytes, we have the possibility to include CLP patient-derived fibroblasts in future studies. Here, we subjected CLP patient-derived keratinocytes to *in vitro* differentiation as well as proliferation assays. We found that the late differentiation markers, LOR and FLG, were significantly less induced in the group of CLP cultures than in the control group upon *in vitro* differentiation. In addition, we discovered various differentiation potentials within the individual CLP keratinocyte cultures and could correlate the terminal differentiation capabilities with the expression levels of the transcription factors IRF6, GRHL3, and KLF4. These data are the first to analyze human keratinocytes isolated from the discarded lip tissue of the orofacial cleft. We anticipate that our findings and the availability of clinical relevant CLP patient-derived cells will stimulate collaborative efforts to gain a better understanding of the genetic and cellular mechanisms involved in the complex pathogenesis of CLP, which could hopefully help in managing CLP individuals in the future.

## Author Contributions

AW and MD performed all the experiments. MD and CK designed the project and wrote the main manuscript. All clinical work was performed by GLS, CC, and IS. All authors critically reviewed the manuscript.

## Conflict of Interest Statement

The authors declare that the research was conducted in the absence of any commercial or financial relationships that could be construed as a potential conflict of interest.

## Footnotes

1 http://www.ncbi.nlm.nih.gov/tools/primer-blast/

2 https://imagej.nih.gov/ij/
