# Supplementary material for: Keratinocytes Isolated From Individual Cleft Lip/Palate Patients Display Variations in Their Differentiation Potential in vitro
Source: Front Physiol. 2018 Nov 29;9:1703. doi: 10.3389/fphys.2018.01703 (PMC6281767; doi:10.3389/fphys.2018.01703)
Supplement: Supplementary file 1 [file Data_Sheet_1.PDF]

Supplementary Material

Keratinocytes Isolated from Individual Cleft Lip/Palate Patients Display Variations in Their Differentiation Potential *In Vitro*

Martin Degen*, Astrid Wiederkehr, Giorgio C La Scala, Christina Carmann, Isabelle Schnyder and Christos Katsaros

*** Correspondence:** Martin Degen: martin.degen@zmk.unibe.ch

1 Supplementary Figures and Tables

1.1 Supplementary Figures

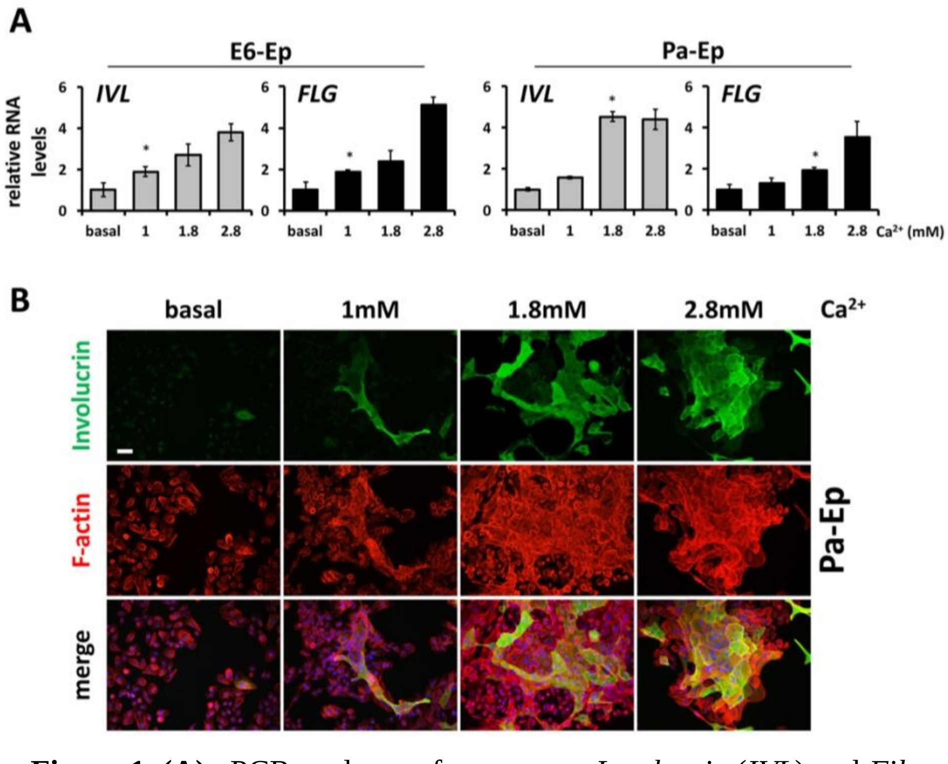

Supplementary Figure 1. (A) qPCR analyses of two genes, *Involucrin* (*IVL*) and *Filaggrin* (*FLG*) in two CLP-keratinocyte strains (E6-Ep and Pa-Ep). Note that the fold induction of both genes after five days in differentiation medium, correlates with the concentration of exogenous Ca^{2+}. Data are expressed as mean ± SEM. n=3. **(B)** Immunofluorescent staining of Pa-Ep (CLP-derived keratinocytes) cultures after five days in differentiation medium stained for Involucrin (green), F-actin (red), and DAPI (blue) confirms the qPCR results. Note that although the highest Ca^{2+} concentration of 2.8 mM resulted in the strongest induction of the two markers, we refrained from this high concentration because we often observed Ca^{2+}-precipitations in our cultures. Scale bar: 50 µm.

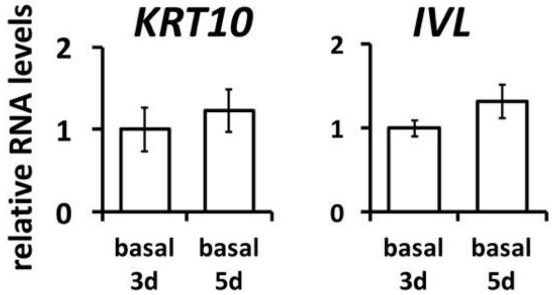

Supplementary Figure 2. qPCR analysis comparing H7-Ep (CLP-derived keratinocytes) cultures grown for three or five days in 0.1 mM KSFM after initial plating shows that there is no significant density-dependent induction of *KRT10* and *IVL* within the 48 h. Data are expressed as mean ± SEM. n=3.

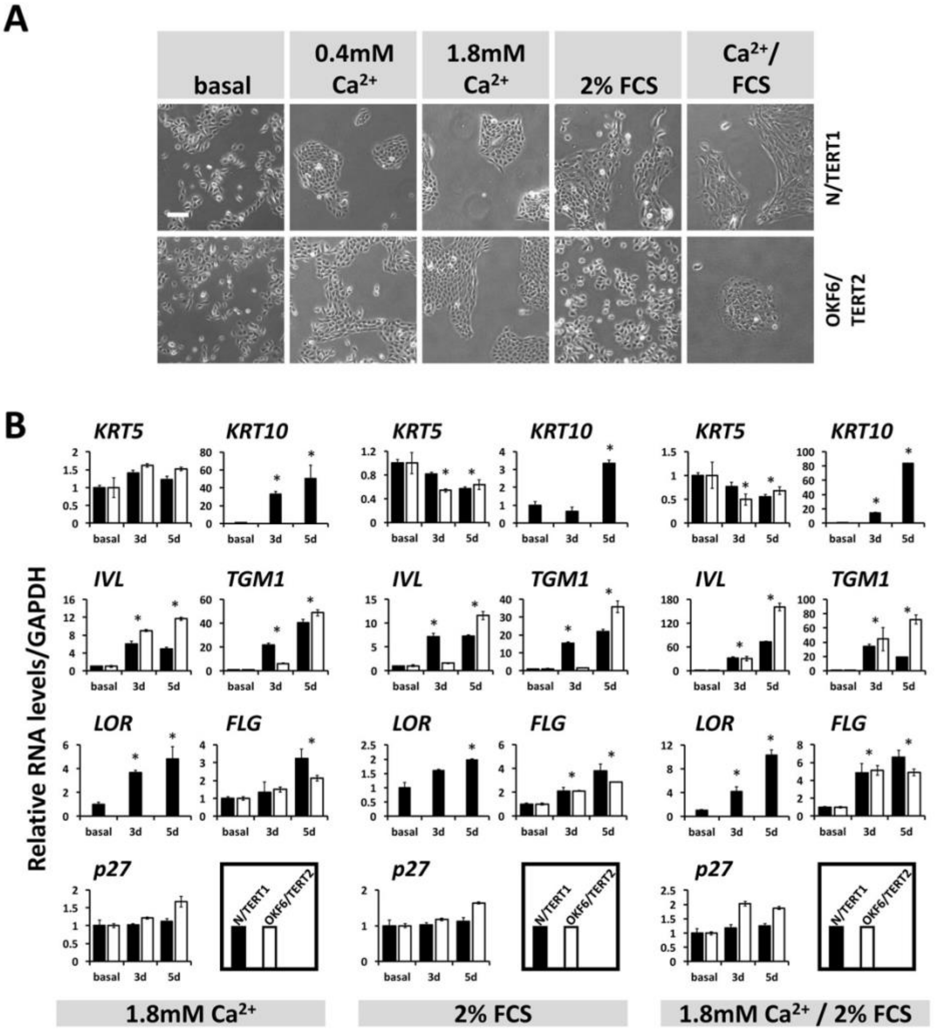

Supplementary Figure 3. (A) Live cell imaging reveals morphological changes of the cells (colonies) upon Ca^{2+} / FCS switch for five days. Scale bar: 200 μm. Note that there are some differences between the morphology of OKF6/TERT2 and N/TERT1 in the presence of 2 % FCS. **(B)** qPCR analyses of various differentiation-related genes in ~ 60 % confluent cultures of N/TERT1 (black bars) and OKF6/TERT2 (white bars) keratinocytes three and five days after induction of differentiation. Genes studied were: 1) marker of basal epidermal layer: *Keratin 5* (*KRT5*); 2) marker of spinal layer: *Keratin 10* (*KRT10*); 3) markers of granular layer: *Involucrin* (*IVL*), *Transglutaminase 1* (*TGM1*), *Loricirin* (*LOR*), *Filaggrin* (*FLG*); 4) marker of cell cycle inhibition: *CDKN1B* (*p27*). mRNA levels have been calculated as described in Methods. Fold induction of mRNA levels is shown compared to the reference levels (basal medium), which has been set to 1. Significance was reached when $p \leq 0.05$ (*) compared to basal level. Note that the oral mucosal keratinocytes (OKF6) do not express detectable basal levels of *KRT10* and *LOR*. Data are expressed as mean ± SEM. n=3.

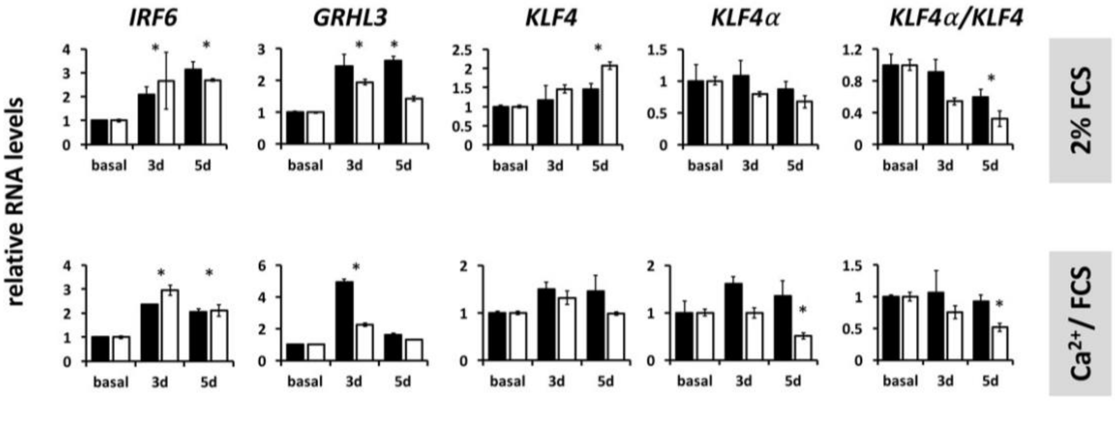

Supplementary Figure 4. qPCR analysis of *IRF6*, *GRHL3*, *KLF4*, and *KLF4α* in ~ 60 % confluent cultures of control keratinocytes (Cx-Ep, black bars) and CLP-keratinocytes (H7-Ep, white bars) three and five days after induction of differentiation with FCS or Ca^{2+}/FCS. mRNA levels have been calculated as described in Materials and Methods. Fold induction of mRNA levels is shown compared to the reference levels (basal medium), which has been set to 1. Significance was reached when $p \leq 0.05$ (*) compared to basal level. Note that the *KLF4α/KLF4* ratio is decreasing upon differentiation. Data are expressed as mean ± SEM. n=3.

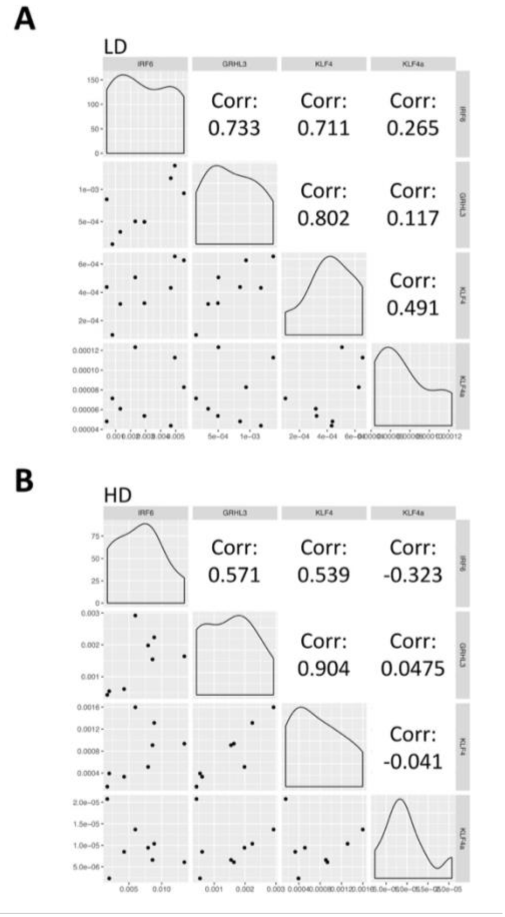

Supplementary Figure 5: Correlations between *IRF6, GRHL3, KLF4,* and *KLF4α.*
(A) Scatter plots, distribution plots, and Pearson's Correlation Coefficient of and between the genes *IRF6, GRHL3, KLF4,* and *KLF4α* at low-density cultures (LD). **(B)** Scatter plots, distribution plots, and Pearson's Correlation Coefficient of and between the same genes at high-density cultures (HD).

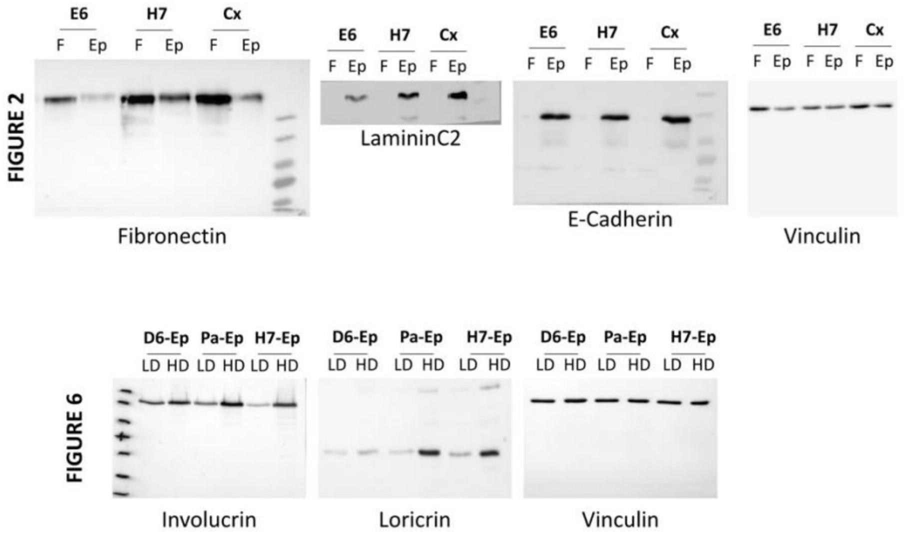

Supplementary Figure 6. Full-length blots of the immunoblot analyses of Figure 2 and Figure 6 are shown. F: Fibroblasts; Ep: Epithelial cells; LD: low-density; HD: high-density.

1.2 Supplementary Tables

HUMAN FORESKIN
EPITHELIAL CELLS (Ep)
FIBROBLASTS (F)

cells	donor age
Cx-Ep	n.a.
Cx-F	
18A-Ep	4y
18A-F	
18B-Ep	7y
18B-F	
18C-Ep	8y
18C-F	
18D-Ep	7y
18D-F	

HUMAN CLEFT LIP
EPITHELIAL CELLS (Ep)
FIBROBLASTS (F)

cells	donor sex	CLP type
Ry-Ep	M	c CLP
Ry-F		unilateral
Pa-Ep	M	i lip
Pa-F		unilateral
M6-Ep	M	i CLP
M6-F		bilateral
A6-Ep	F	i CLP
A6-F		unilateral
B6-Ep	M	c CLP
B6-F		bilateral
C6-Ep	F	c CLP
C6-F		bilateral
D6-Ep	M	c CLP
D6-F		bilateral
E6-Ep	M	i CLP
E6-F		unilateral
F6-Ep	F	c CLP
F6-F		unilateral
G6-Ep	M	c CLP
G6-F		unilateral
H7-Ep	M	c CLP
H7-F		unilateral
I7-Ep	F	c CLP
I7-F		unilateral
J7-Ep	M	c CLP
J7-F		unilateral
K7-Ep	M	i lip
K7-F		unilateral
L7-Ep	F	c CLP
L7-F		unilateral
M7-Ep	M	i lip
M7-F		unilateral
N7-Ep	M	c CLP
N7-F		bilateral
O8-Ep	M	c CLP
O8-F		unilateral
P8-Ep	M	c CLP
P8-F		bilateral
Q8-Ep	M	i lip
Q8-F		unilateral
R8-Ep	M	i lip
R8-F		unilateral
S8-Ep	M	i CLP
S8-F		unilateral
T8-Ep	F	c CLP
T8-F		unilateral

Supplementary Table 1. Total list of our growing cell bank. Cell strain names and donor ages are indicated for the foreskin-derived cells (left), and cell strain names, donor sex, as well as CLP type is indicated for the CLP patient-derived cells. Ep: epithelial cells; F: fibroblasts; y: years; M: male; F: female; n.a.: not available; c: complete; i: incomplete; VWS: van der Woude Syndrome.

GENE	PRIMER SEQUENCE FORWARD (5'-3')	PRIMER SEQUENCE REVERSE (5'-3')	amplicon size (bp)
Vimentin (VIM)	TGTCCAAATCGATGTGGATGTTTC	TTGTACCATTCTTCTGCCTCCTG	117
Fibronectin (FN)	CCATTATTGGGTACCGCATCACA	AGGAGGAACAGCCGTTTGTTGT	200
**Tenascin-C (TNC)**	TCAAAGACGTGCCAGGAGAC	TCTGTCTGGGAAACACGTCG	202
Lamininγ2 (LAMC2)	CTCTGCTTCTCGCTCCTCC	TCTGTGAAGTTCCCGATCAA	108
E-Cadherin (CDH1)	AGAACGCATTGCCACATACACT	TCTGATCGGTTACCGTGATCAA	101
Keratin 4 (KRT4)	CAACCTGAAGAACACCAAGA	AAGAGTCTGGCACTGCTTC	100
Keratin 5 (KRT5)	CTGCTGGAGGGCGAGGAATGC	CCACCGAGGCCACCGCCATA	125
Keratin 10 (KRT10)	TGGTTCAATGAAAAGAGCAAGGA	GGGATTGTTTCAAGGCCAGTT	151
Keratin 13 (KRT13)	CTGAACAAGGAGGTGTCTACCA	ATAGCGGCACTCCGTCTCT	162
Keratin 14 (KRT14)	GGCCTGCTGAGATCAAAGACTAC	CACTGTGGCTGTGAGAATCTTGTT	80
Keratin 19 (KRT19)	TGAGTGACATGCGAAGCCAAT	ACCTCCCGGTTCAATTCTTCA	100
Involucrin (IVL)	GGCCCTCAGATCGTCTCATA	CACCCTCACCCCATTAAAGA	131
Transglutaminase 1 (TGM1)	CCCCCGCAATGAGATCTACA	ATCCTCATGGTCCACGTACACA	73
Filaggrin (FLG)	CTGGACACTCAGGTTCCCAT	TTTCGTGTTTGTCTGCTTGC	103
Loricrin (LOR)	AGACCCAGCAGAAGCAGGCG	AGCAGAACTAGATGCAGCCG	200
Interferon Regulatory Factor 6 (IRF6)	GCTCTTCCATATCATGGCCCTC	CTACAGCCCAGGCCTTAAAAA	200
Grainyhead-like 3 factor (GRHL3)	CCCCCATGTCCAGAGGACTA	ACTCGCCTGACTTGATGTGG	91
Krüppel-like factor 4 (KLF4)	GAGAAGACACTGCGTCAAGC	AGTCGCTTCATGTGGGAGA	71
Krüppel-like factor 4α (KLF4α)	GTGCCCCGAATAACAGCTCA	ACGATCGTCTTCCCCTCTTT	77
p27Kip1 (CDKN1B)	CCGGTGGACCACGAAGAGT	GCTCGCCTCTTCCATGTCTC	66
Proliferating-Cell-Nuclear-Antigen (PCNA)	CGACACCTACCGCTGCGACC	TAGCGCCAAGGTATCCGCGT	133
Marker of proliferation Ki-67 (Ki-67)	TGACTTCCTTCCATTCTGAAGAC	TGGGTCTGTTATTGATGAGCC	109
Glyceraldehyde 3-phosphate dehydrogenase (GAPDH)	CTCTGACTTCAACAGCGACACCC	TCCTCTTGTGCTCTTGCTGGGGC	199

Supplementary Table 2. Sequence of the qPCR primers used in this study. bp: base pairs
